# Convulsions

**Published:** 1887-04

**Authors:** 


**Convulsions.**—Convulsions may frequently be cut short, like magic, by turning the patient on his left side. The nausea as an after effect of chloroform or ether narcosis may generally be controlled in the same manner.—*Peoria Medical Monthly.*

